# Women and the risk of Alzheimer's disease

**DOI:** 10.3389/fgwh.2023.1324522

**Published:** 2024-01-05

**Authors:** Mary A. O’Neal

**Affiliations:** Department of Neurology, Brigham and Women's Hospital, Harvard Medical School, Boston, MA, United States

**Keywords:** women, Alzheimer’s disease—AD, sex, risk factor, gender

## Abstract

**Purpose of the review:**

This review will elucidate reasons to explain why women may be at greater risk for Alzheimer's disease.

**Recent findings:**

Potential mechanisms to explain sex and gender differences in Alzheimer dementia include: differences in risk associated with the apolipoprotein E 4 allele; telomere shortening- which is linked with neurodegeneration, higher incidence of depression and insomnia in women as psychiatric co-morbidities which are linked with an increased Alzheimer disease risk, disorders of pregnancy including gestational hypertension and preeclampsia and psychosocial factors such as educational level which may contribute to differences in cognitive reserve.

**Summary:**

The sex and gender differences in Alzheimer's disease can be explained by biological and psychosocial factors.

## Introduction

Alzheimer's disease (AD) is the most common cause of dementia affecting more than 5.5 million Americans, two thirds of whom are women (1). Age is a known risk factor for development of AD, with the risk doubling for each decade after age 60. It is a well-known fact that women live longer, thereby explaining the difference in prevalence of the disease. Incidence studies examining sex differences in AD are equivocal. A majority of studies do not demonstrate any sex differences in the incidence of AD. See Table 1 (2–8) A meta-analysis of seven population -based studies looking at the incidence of AD found that the increase in the incidence rate slows after age 85. In contrast to this age effect, the meta-analysis showed a significant effect of sex, where the odds ratio of women developing AD compared to men was 1.56 (9). The Framingham study suggests that the difference in disease prevalence is due to a “survivor bias”, as men who survive beyond age 65 may have lower cardiovascular risk factors which may explain the lower risk of dementia compared to women after the age of 80 years (10).

**Table 1:** Summary of studies 1975–2013.

Study location, type	Years	Incidence rates to develop AD women/men
Rochester, MN; Retrospective	1975–1984	Same
Framingham, MA; Prospective	1976–1978 to 1984–1985	Same
Rural area of southwestern Pennsylvania; Prospective	1987–1989 to 1998	Same
Baltimore, MD Prospective	1/1985–5/1998	Insignificant trend towards women having higher incidence rates
East Boston, MA Prospective	1982–12/1992	Same
Seattle, WA Prospective	1994–96 to 1996–98	Same
Cache County, UT Prospective	1995–96 to 1998–99	Greater incidence in women than in men after age 85.
England, Wales- 2 studies Prospective	Study 1 1990–93 to 1993–95 Study 2 2008–11 to 2011–13	Study 1-Women had lower incidence than men Study 2 - Same

There are multiple potential biological mechanisms that may explain the sex and gender differences in AD. These include differences in genetic risk, response to aging, hormonal effects, psychiatric and pregnancy co-morbidities as well as lifestyle/psychosocial factors effecting cognitive reserve. This review will explore these mechanisms.

## Apolipoprotein E4-genetic risk

The apolipoprotein E4 (APOE4) allele is the most potent genetic risk factor for late onset sporadic AD. The APOE4 allele generates a dose dependent risk of developing AD, where patients with the E4/E4 genotype have an increased risk of AD compared to the E4/E3 genotype (11). The apolipoprotein E (APOE) protein is widely distributed throughout the human body. In the brain, astrocytes primarily produce APOE (12). The APOE protein in AD plays an important role in amyloid-beta protein transcription, production, aggregation and clearance (13).

The risk of developing AD related to the APOE4 allele effects both sexes equally. However, women who carry the APOE4 allele are more likely to develop mild cognitive impairment (MCI) than men. In addition, among patients with MCI, women with either the *APOE3/3* or *APOE3/4* genotypes, are more likely to develop AD compared to men (14). In a meta-analysis of 27 studies with 58,000 participants, looking at patients with 1 copy of APOE4 allele, women were at a fourfold increased risk to develop AD at younger ages, 65–75 years (15). Further, women carriers had increased total tau in cerebrospinal fluid, a biomarker in AD indicative of neuronal degeneration (16). However, these studies may be confounded by the fact that they did not control for educational level. A study which looked at the longitudinal rates of change from baseline in 398 MCI patients showed women with MCI had greater rates of cognitive and functional progression than men. The effect was greater in APOE4 carriers. In this study educational attainment was statistically greater in men, but the difference was small (17). Available data suggests that the APOE4 may modulate the risk of AD in a sex specific manner.

## Telomere shortening and aging

Telomeres are the DNA structures that cap the ends of chromosomes. They are important to protect chromosomes from degradation. Telomere shortening has been associated with limited stem cell function, regeneration and organ maintenance in aging. The enzyme telomerase offsets this reaction by adding repeat telomeres to the terminal DNA. A reduced telomere length is associated with AD especially in females (18). Evidence demonstrates that APOE4 carriers have shorter leukocyte telomere lengths compared to noncarriers (19). This supports the idea that the APOE4 carriers undergo premature aging.

Telomere length demonstrate significant sex differences. In adulthood, women have significantly longer telomere length than men of the same age and this effect appears to be driven by estrogen. Estrogen both increases telomerase activity and decreases oxidative stress (20). In an interesting study, conducted over two years, healthy postmenopausal women who were APOE4 carriers showed significant leucocyte telomere shortening compared to noncarriers. Further the APOE4 carriers who remained on hormonal replacement therapy did not show telomere attrition and this effect was not seen in the noncarriers. Thus, suggesting that hormonal therapy might modulate AD risk for those who are vulnerable (21). Another explanation for this difference may be related to sex differences in educational attainment as this appears to have a protective effect against telomere shortening (22).

## Hormonal effects

Initial studies from Cache county Utah supported the idea that there may be a “window of opportunity” for hormonal therapy on cognition. In a population- based study of over 2,000 nondemented women over age 65 (when the covariates of lower education, depression, and *APOE ε*4 status were controlled) lifetime hormonal replacement therapy (HRT) use was associated with a better baseline mini-mental status exam scores and a slower rate of cognitive decline (23). In another prospective study of 1,889 women from Cache county Utah to look at incidence of dementia those who used HRT had a reduced risk of AD compared with non-HRT users (adjusted HR, 0.59; 95% CI, 0.36–0.96). Risk varied with duration of HRT use, such that the sex-specific increase for women disappeared with more than 10 years of use (24). It appears from these studies that the beneficial effect of HRT is dependent on the timing. However, the generalizability of these studies is unclear as both were performed in a single county and educational and socioeconomic factors may have influenced those who could participate.

In 2003, a large randomized controlled trial, The Women's Health Initiative Memory study (WHIMS) showed that postmenopausal women, ages 65–79, who had not had a hysterectomy, when treated with estrogen and progesterone compared to controls had a doubled risk of dementia. The increased risk of dementia in women treated with hormonal therapy would result in 23 new cases of dementia per 10,000 women/year (25). This well-designed trial clearly demonstrated the negative effects of postmenopausal hormone replacement on cognition.

There have been mixed results in studies to see if there is indeed a therapeutic window for HRT. A prospective cohort study showed that women who used any type of HRT within 5 years of menopause had a 30% less risk of AD. This benefit was also realized in women who used HRT more than 10 years after menopause (26). A 20-year prospective cohort study from Finland following women ages 47–56 did not provide strong evidence that postmenopausal hormone replacement therapy prevents AD. Although, a protective association between long-term (>10 years) self-reported use of HT and AD was observed. This finding indirectly supports the effectiveness of HT if started in the early postmenopausal period (26).

A recent retrospective population- based study demonstrated that women with 5 or more pregnancies had 1.7-fold increased risk of AD compared with women with 1–4 completed pregnancies. In addition, women who had incomplete pregnancies showed half the level of AD risk compared with those who never experienced an incomplete pregnancy (27). Whether these findings are related to hormonal changes, differences in medical comorbidities or education/socioeconomic status remains to be seen.

## Pregnancy complications

Retrospective studies have shown that women who had preeclampsia have higher stroke risks even decades later. See Table 2 Pregnancy related complications unmasks those women at risk for cerebrovascular complications. The increased risk of stroke put these women at risk for vascular dementia and increase risks of cognitive decline. Another study documented those women who had hypertensive disorders of pregnancy had an increased risk of cognitive problems primarily associated with poorer working memory and verbal learning 15 years after pregnancy (28).

**Table 2:** Preeclampsia and stroke in later life.

Study date	Total no. of subjects	Design	OR-95%CI
Lykke-2009	782,287	Retrospective cohort	1.36–1.66 (1.29–2.14)
Funai-2005	37,061	Retrospective cohort	3.07 (2.18–4.33)
Kestenbaum-2003	124,141	Case control study	2.53 (1.70–3.77)
Irgens-2001	626.272	Retrospective cohort	Preterm preeclampsia 5.0 (2.09–12.35)

Vascular dementia can be caused by either a strategically placed stroke or from small vessel disease. The typical cognitive problems found in patients with vascular dementia are slowed processing speed, impairments in executive function and visual memory, whereas verbal learning is not as affected. This is different from the results of the hypertensive disorder of pregnancy study which showed worsened working memory and verbal learning. Problems which are more in those with seen in with AD. Potentially pregnancy complications may impact cognition by contributing to a mixed dementia. Mixed dementia from both AD and vascular causes is common with small vessel disease of different types underpinning both etiologies (29).

## Psychiatric co-morbidities-depression and insomnia

Depression increases the risk of AD and women have twice the risk of depression compared to men (30). There is evidence that early life depression can act as a risk factor for later life dementia, and that depression later in life may be a prodromal feature to dementia (31). A meta-analysis of available studies showed a positive correlation between the length of time after a diagnosis of depression and the risk of developing AD, suggesting that depression is a risk factor for AD (30).

In the WHIMS study women with depression were almost twice as likely to develop MCI and AD (32). The risk of developing AD appears to be related to both the severity and timing of the depression. For example, one study demonstrated that patients followed for a mean of 27 months with MCI and active depression within the last 2 years had a 41.7% conversion to AD as compared to 31.6% conversion to AD in patients with a more remote history of depression (33).

Insomnia may also be a risk factor for accelerated cognitive decline and AD. Cognitive ability is sleep dependent, especially memory consolidation. Several longitudinal studies confirmed that patients with insomnia were twice as likely to have cognitive decline or a diagnosis of AD (34, 35). Women have a higher prevalence of insomnia. It is not clear if insomnia is an independent risk factor for AD or linked due to its interplay with stress and depression.

## Educational status-cognitive reserve

Lower education levels and occupational attainment pose similar risk for AD in both women and men. Women, older than 65 years of age, have had fewer opportunities for higher education and professional achievement, directly affecting their cognitive resilience, thus putting them at risk. Recent population trends indicate that the education gap between women and men is in decline with more women in the workplace. In addition, women are achieving both greater professional success and financial status (36).

## Conclusions

Women bear a larger burden of the Alzheimer's disease epidemic. The difference in risks for this disorder can be explained by sex and gender specific variances in genetics, response to aging, hormonal influences, psychiatric conditions as well as psychosocial factors.

## Take Home Points

1. Alzheimer's disease is more prevalent in women
2. The risks associated with the APOE allele are stronger in women
3. There are sex differences in how telomeres respond to aging and hormonal changes
4. There may be a beneficial window where estrogen exposure improves cognition for women at risk for AD
5. Hypertensive disorders of pregnancy can contribute to risks of dementia
6. Gender differences in psychiatric co-morbidities especially depression as well as educational status may also impact AD risk
